# Clinical Outcomes of Computational Virtual Mapping-Guided Catheter Ablation in Patients With Persistent Atrial Fibrillation: A Multicenter Prospective Randomized Clinical Trial

**DOI:** 10.3389/fcvm.2021.772665

**Published:** 2021-12-08

**Authors:** Yong-Soo Baek, Oh-Seok Kwon, Byounghyun Lim, Song-Yi Yang, Je-Wook Park, Hee Tae Yu, Tae-Hoon Kim, Jae-Sun Uhm, Boyoung Joung, Dae-Hyeok Kim, Moon-Hyoung Lee, Junbeom Park, Hui-Nam Pak, the CUVIA-AF 2 Investigators

**Affiliations:** ^1^Inha University College of Medicine and Inha University Hospital, Incheon, South Korea; ^2^Division of Cardiology, Department of Internal Medicine, Yonsei University Health System, Seoul, South Korea; ^3^Division of Cardiology, Department of Internal Medicine, Ewha Womans University, Seoul, South Korea

**Keywords:** catheter ablation, computational modeling, recurrence, dominant frequency, atrial fibrillation

## Abstract

**Background:** Clinical recurrence after atrial fibrillation catheter ablation (AFCA) still remains high in patients with persistent AF (PeAF). We investigated whether an extra-pulmonary vein (PV) ablation targeting the dominant frequency (DF) extracted from electroanatomical map–integrated AF computational modeling improves the AFCA rhythm outcome in patients with PeAF.

**Methods:** In this open-label, randomized, multi-center, controlled trial, 170 patients with PeAF were randomized at a 1:1 ratio to the computational modeling-guided virtual DF (V-DF) ablation and empirical PV isolation (E-PVI) groups. We generated a virtual dominant frequency (DF) map based on the atrial substrate map obtained during the clinical AF ablation procedure using computational modeling. This simulation was possible within the time of the PVI procedure. V-DF group underwent extra-PV V-DF ablation in addition to PVI, but DF information was not notified to the operators from the core lab in the E-PVI group.

**Results:** After a mean follow-up period of 16.3 ± 5.3 months, the clinical recurrence rate was significantly lower in the V-DF than with E-PVI group (*P* = 0.018, log-rank). Recurrences appearing as atrial tachycardias (*P* = 0.145) and the cardioversion rates (*P* = 0.362) did not significantly differ between the groups. At the final follow-up, sinus rhythm was maintained without any AADs in 74.7% in the V-DF group and 48.2% in the E-PVI group (*P* < 0.001). No significant difference was found in the major complication rates (*P* = 0.489) or total procedure time (*P* = 0.513) between the groups. The V-DF ablation was independently associated with a reduced AF recurrence after AFCA [hazard ratio: 0.51 (95% confidence interval: 0.30–0.88); *P* = 0.016].

**Conclusions:** The computational modeling-guided V-DF ablation improved the rhythm outcome of AFCA in patients with PeAF.

**Clinical Trial Registration:** Clinical Research Information Service, CRIS identifier: KCT0003613.

## Introduction

Atrial fibrillation (AF) is the most common arrhythmia in clinical practice and has been associated with increased risks of heart failure, strokes, dementia, and cardiovascular death (1). AF catheter ablation (AFCA) is an effective therapy in patients with symptomatic and drug-refractory AF. Because AF is a chronic progressive disease with an annual progression rate of 7.2–15%, appropriate rhythm control by AFCA reduces the heart failure mortality, overall mortality, and hospitalization rates as well as the stroke risk and improves the cognitive function and renal function (2–4). However, clinical long-term AF recurrence after AFCA still remains high in patients with persistent AF (PeAF) and longstanding PeAF (5). This may be because the atrial substrate changes during AF progression and extrapulmonary vein (PV) foci play important roles in AF recurrence after the PeAF ablation (6). Nevertheless, no single empirical extra-PV ablation strategy, such as a linear, electrogram-guided, low voltage–guided, or rotor ablation, has been proven to improve the rhythm outcome of AFCA in patients with non-paroxysmal AF (7–9). In other words, the use of the current sequential atrial substrate mapping technology with a multielectrode catheter is not adequate to find the AF driver, as in contrast to the remarkable improvements made in the catheter technology for a long-lasting PV isolation (PVI). Recently, computational modeling has demonstrated the potential applicability in cardiac arrhythmia interventions (10, 11). Computational modeling allows for high-density, entire-chamber mapping while reflecting the personalized atrial anatomy and physiology (12). It enables a mechanism-based virtual ablation test targeting various AF wave-dynamic parameters and the prediction of the clinical outcomes by a reproducible condition control (10, 11, 13, 14). We previously reported the clinical feasibility and effectiveness of AF ablation lesion sets chosen using an *in silico* ablation relative to that of empirically chosen ablation lesion sets in patients with PeAF in a multicenter prospective clinical study (10, 11). In this study, we improved the existing computational modeling software (CUVIA version 2.5; Laonmed Inc., Seoul, Korea), which enabled one to conduct an entire-chamber mapping of the AF drivers based on the acquired substrate map during atrial pacing. We designed this multicenter prospective randomized clinical trial (RCT) by collaboration between the clinical ablation team and simulation team in real-time. Under this arrangement, the operator acquired the atrial substrate map and sent the data to the simulation team, and then the operator proceeded with the PVI and received the outcome of the simulation for the extra-PV targets after the PVI procedure. We compared the outcomes of the real-time computational modeling–guided extra-PV target ablation and empirical AFCA in patients with non-paroxysmal AF.

## Materials and Methods

### Study Design and Population

**Figure 1:**
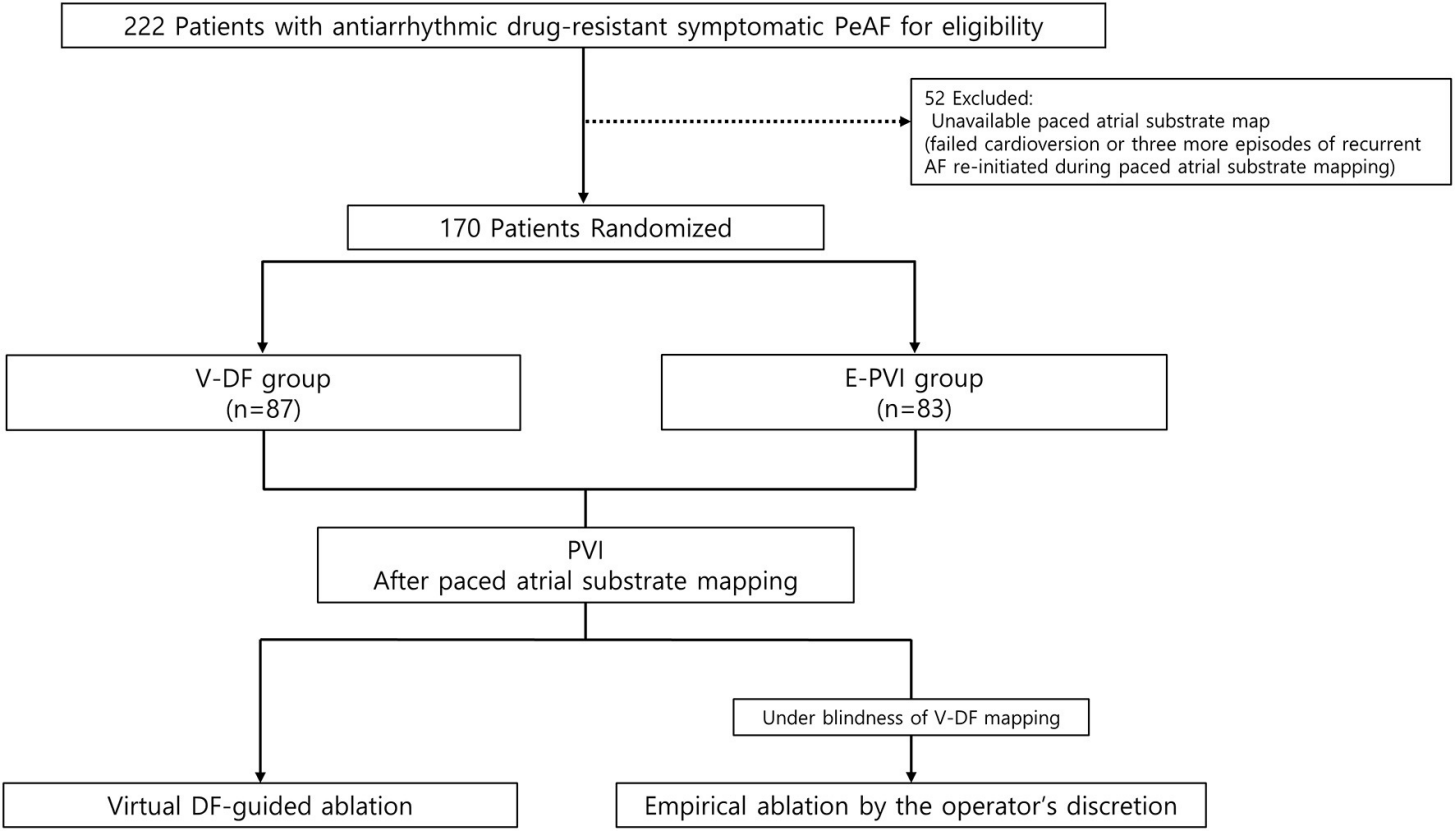
Study flow diagram. The enrolled patients were randomly assigned to either the computational modeling–guided or empirical ablation groups. PeAF, persistent atrial fibrillation; RFCA, radiofrequency catheter ablation.

This randomized, open-label, multicenter trial included drug-refractory AF patients undergoing AFCA at three tertiary hospitals in Korea (Clinical Research Information Service, CRIS identifier: KCT0003613). The study protocol was approved by the Institutional Review Board of each participating center and complied with the principles of the Declaration of Helsinki. All participants provided written informed consent before study enrollment. Figure 1 shows the study design of the CUVIA-AF2 study. We enrolled a total of 222 patients with AAD-resistant symptomatic PeAF undergoing catheter ablation. The key exclusion criteria were as follows: (1) an age younger than 20 or older than 80 years, (2) paroxysmal AF, (3) valvular AF, (4) significant structural heart disease other than left ventricular hypertrophy, (5) left atrial (LA) diameter >55 mm, (6) history of AF ablation or cardiac surgery, and (7) the LA voltage map was not available due to recurrent (>3 episodes) or re-initiated AF after cardioversion. In patients with sustaining AF at the beginning of the procedure, we performed internal cardioversion by utilizing biphasic shock (2–20 J) with R wave synchronization (Lifepak12, Physiocontrol Ltd., Redmond, WA, USA) to acquire the LA substrate map. If the cardioversion failed or AF recurred during substrate mapping, we repeated cardioversion without using an antiarrhythmic drug at least 3 times. However, among enrolled a total of 222 patients, 52 (23.4%) were excluded due to failed internal cardioversion or >3 episodes of recurrent AF re-initiated during paced atrial substrate mapping, which provided the mandatory electrophysiologic data for our realistic computation modeling. All patients were treated with antiarrhythmic drugs (AADs) at baseline before AFCA. All antiarrhythmic drugs (AADs) were discontinued for at least five half-lives, and amiodarone was stopped at least 4 weeks before the procedure. Finally, 170 patients (70.6% men; 59.2 ± 11.3 years) were randomly assigned, using a random number table, to the virtual dominant frequency (DF) map-guided catheter ablation (V-DF, 87 patients) and empirical PVI ablation (E-PVI, 83 patients) groups.

### CUVIA-AF2 Study Protocol

**Figure 2:**
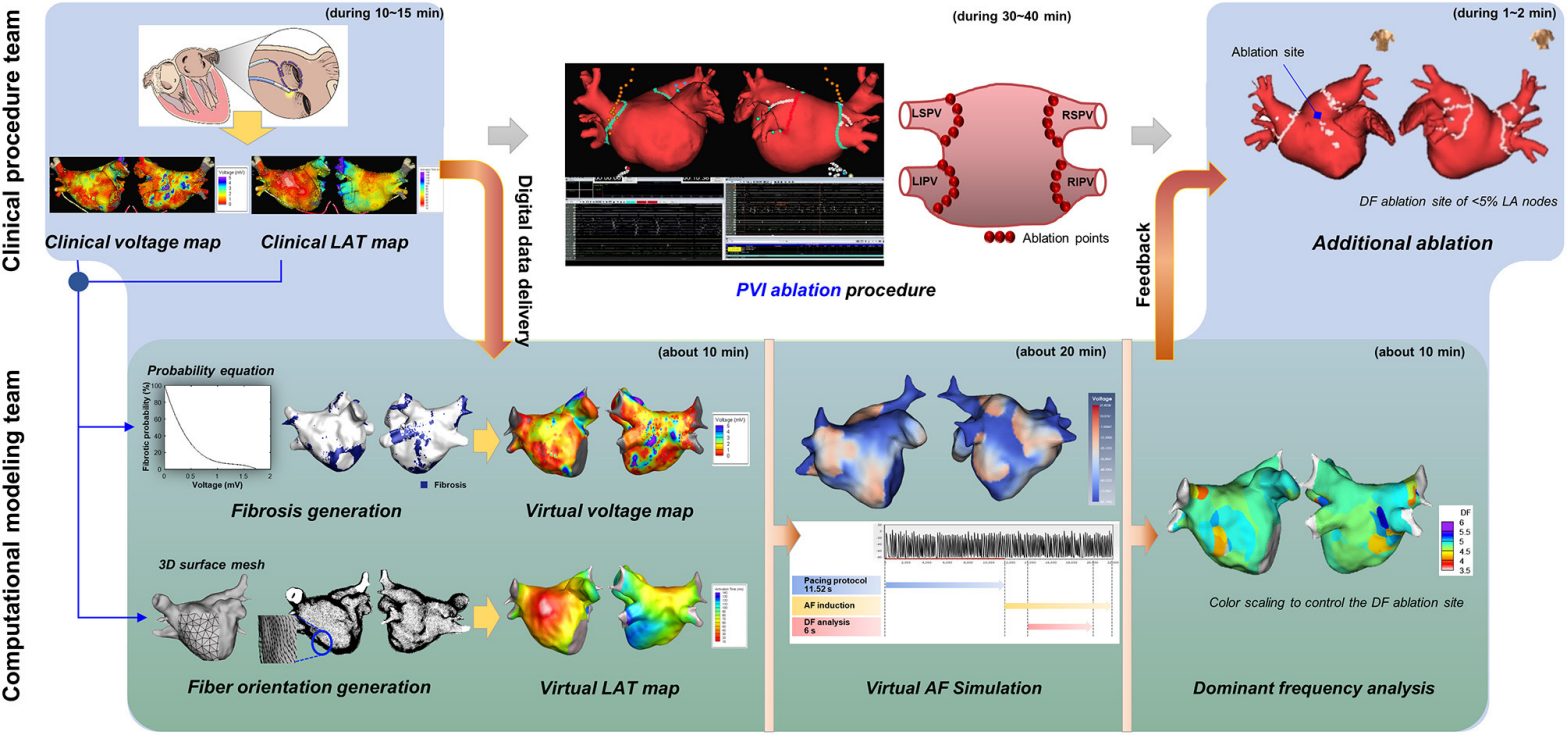
CUVIA-AF 2 approach flowchart.

Figure 2 shows the study process for the CUVIA-AF2 as a real-time collaborative protocol between the clinical procedure and computational modeling teams. At the beginning of the AFCA procedure, the clinical procedure team reconstructed the LA substrate map (bipolar voltage and local activation maps) using EnSite™ NavX™ acquired with a multielectrode catheter (AFocus, Abbott, Chicago, IL, USA) during high right atrial pacing with a cycle length of 500 ms. After extracting and transferring the digital data of the atrial substrate map to the computational modeling team, the clinical operator concentrated on conducting the PVI procedure. The substrate mapping data were analyzed in an on-site procedure room or transferred through the in-hospital network. During the 30–40 min PVI procedure, the modeling team conducted a “virtual AF” induction and DF analysis. “Virtual AF” means AF simulation induced by ramp pacing on the LA substrate map in the computational modeling. This collaborative approach was conducted real-time, and it took <10 min for the integration of the clinical substrate mapping data into the human atrial cell model, including the personalized fibrosis and fiber orientation, about 20 min for inducing and maintaining virtual AF; and <10 min for the virtual DF analysis (11, 15). The computational modeling team provided the analyzed DF color map to the operator in the V-DF group but not in the E-PVI group. The operator then completed additional ablation of DF areas in the V-DF group, but no additional DF ablation was performed in the E-PVI group. However, 14.5% of the E-PVI group underwent an extra-PV ablation in addition to the PVI per the operators' discretion, mostly due to the existence of extra-PV triggers.

### Computational Modeling of the AF and DF Analyses

We developed CUVIA version 2.5 for use in real-time AFCA procedures using the computed unified device architecture (CUDA) platform. The CUDA-based design methods and performance have been demonstrated in previous studies (12). The LA geometry was reconstructed with the substrate mapping points provided by the clinical procedure team. The electro-anatomical mapping procedure to acquire approximately over 1,000 mapping points in each patient took ~10–15 min. Since there was a physical and temporal limitation to the number of mapping points acquired by the operator, we generated and refined the reconstructed high-resolution mesh using the CUVIA software to improve the accuracy of the simulation. The final number of nodes was set at 400,000–500,000 and the adjacent length between the two nodes was ~300 μm. The LA mesh included the LA appendage and myocardial sleeves of the PV for realistic implementation (16). In the LA mesh, the opening area of the mitral valve and PV vessel was set as a non-conductive area. Then, the personalized fibrosis and fiber orientation were calculated with the clinical substrate mapping data and integrated into the LA mesh node of the human atrial cell model (12).

The cellular ionic currents were calculated based on the modified Courtemanche human atrial model, and electrical wave propagation was simulated with the monodomain equation (12). The following equation was used for the computational modeling of the electric wave propagation on the LA wall (15):


(1) $$\begin{array}{r} \frac{\partial V_{m}}{\partial t}=\frac{1}{\beta C_{m}}\left\{\nabla \cdot D\nabla V_{m}-\beta \left(I_{ion}+I_{s}\right)\right\}, \end{array}$$

where *Vm* (volt) is the membrane potential; β (meter^−1^) is the membrane surface-to-volume ratio; *Cm* (farad/meter^2^) is the membrane capacitance per unit area; *D* (siemens/meter) is the conductivity tensor; and *I*_*ion*_ and *I*_*s*_ (ampere/meter^2^) are the ion and stimulation currents, respectively, the above equation was adopted in parallel on the graphics processing unit as a CUDA kernel using a generalized finite difference system to simulate the electrical wave propagation (15). For the ionic remodeling, the fibrotic cells were initialized in the AF remodeled state and non-fibrotic cells were initialized in the normal state (17, 18). When compared to non-fibrotic cells, the I_to_, I_kur_, I_CaL_, and I_K1_ currents of fibrotic cells changed to −70, −50, −70, and +111%, respectively (Supplementary Table 1). The pacing location for AF induction was estimated from the clinical local activation map, and reentry was initiated by rapid pacing. The pacing intervals were set from 200 to 120 ms (total of eight cycles in 10-ms intervals), and the total pacing time was set to 11,520 ms (19). We observed the AF maintenance for 22.5 s and measured the DF values for 6 s after the AF induction. The DF was analyzed as the frequency of the maximum peak power as described in previous research (20). We ablated the total area of the virtual DF sites in the V-DF group, but the DF ablation sites were adjusted to <5% of the LA nodes by color scaling and then delivered to the clinical procedure team. This approach was based on the previous work that ablation in an area of <5% of the critical mass does not change the termination or defragmentation rates (13). The no-flux condition was applied for all boundaries.

### Definition of the Fiber Orientation From Atlases

We used an atlases-based mesh from each patient's left atrial (LA) geometry (21, 22) to describe the fiber orientation and performed high-density and entire-chamber atrial fibrillation (AF) mapping using personalized electrophysiological mapping data. The vector of the fiber orientation was generated at each node of the LA mesh along the myocardial fiber direction, and the conduction difference according to the orientation was realized by the fiber tracking method (12). The fiber orientation was adjusted based on the clinical local activation time map. The conductivity in the direction perpendicular to the vector was smaller than the conductivity in the vector direction. The conductivity of the model was applied at 0.1264 S/m (non-fibrotic longitudinal cell), 0.0546 S/m (fibrotic longitudinal cell), 0.0252 S/m (non-fibrotic transverse cell), and 0.0068 S/m (fibrotic transverse cell) (23). This procedure was performed at high speed using graphics processing unit-based software.

### Determination of Fibrotic Cells Based on the Clinical Voltage Maps

The fibrosis regions were determined based on a clinically acquired bipolar voltage map. First, the LA mesh was reconstructed with each patient's clinical substrate mapping data, and the clinical bipolar voltage was interpolated into the 3D LA model using the nearest neighbor mapping. To determine the fibrosis or non-fibrosis at each node, we calculated the fibrosis probability through the following equation with the clinical bipolar voltage (24):


(2) $$P_{fibrosis}\;=\{\begin{array}{c} \;1,\;X<0\\ -40.0X^{3}+155X^{2}-206X+99.8,\;0\leq X\leq 1.74\\ \;0,\;1.74<X \end{array}$$

where *P*_*fibrosis*_ is the probability of fibrosis at a given node and *X* is the bipolar voltage at that node. If *X* is greater than the cutoff value of 1.74 mV, *P*_*fibrosis*_ converges to zero. It was developed by comparing the predicted fibrosis rates in a 3D atrial model with pre- and post-ablation fibrosis data. As a result, fibrosis was determined with a probability of fibrosis between 0 and 1 calculated based on clinically acquired bipolar voltage data at each node.

### AFCA

**Figure 3:**
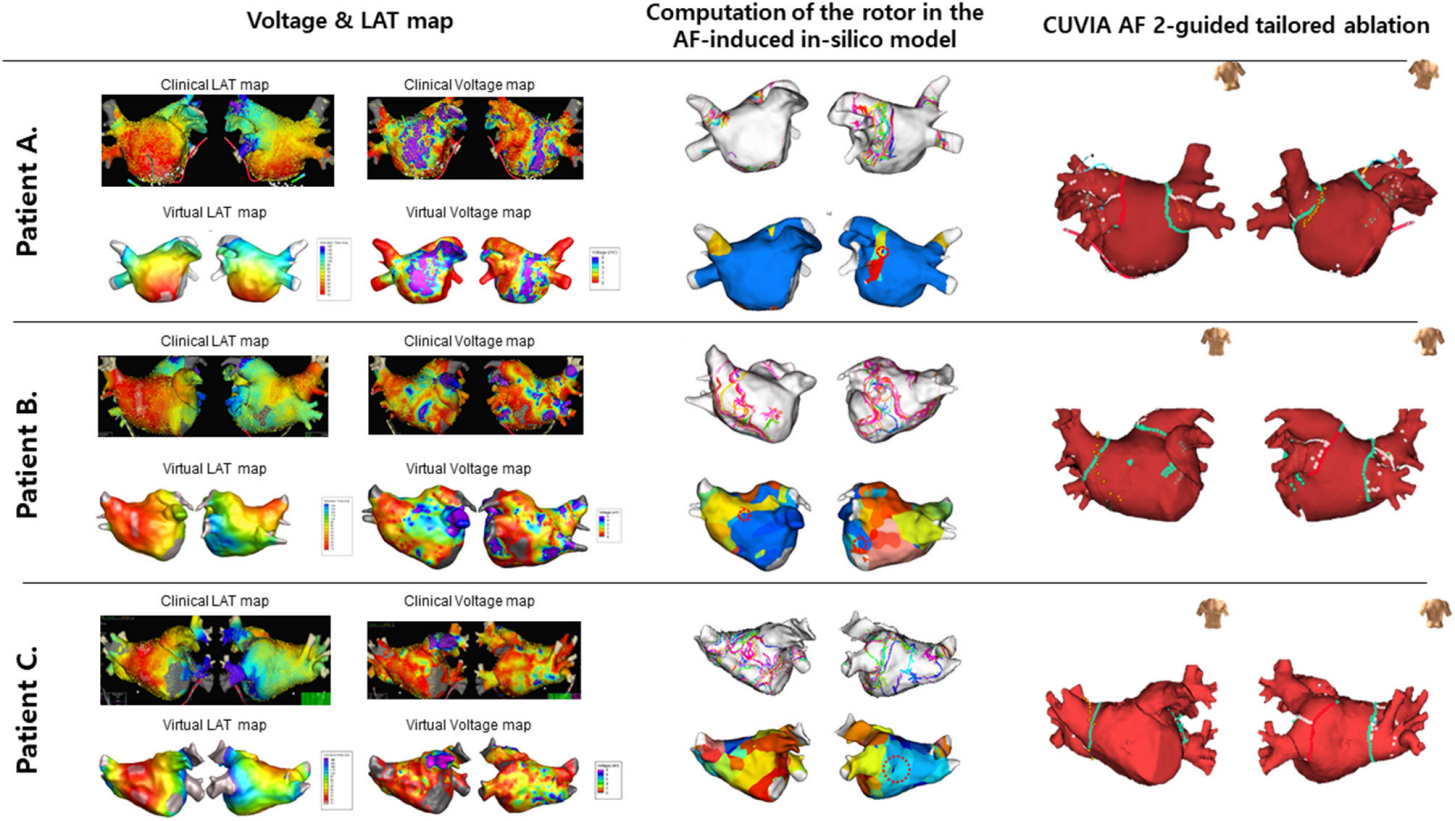
Examples of the process to determine the CUVIA-AF ablation targets in three patients. **(A)** A 52-year-old man with DF located in the left inferior PV in the V-DF group. **(B)** A 56-year-old man with multiple DFs located on the anterior and posterior walls in the V-DF group. **(C)** A 72-year-old man with a DF located on the roof in the E-PVI group.

Electrophysiological mapping and AFCA have been described previously (11). Briefly, we used an open irrigated-tip catheter or a contract-force ablation catheter to deliver radiofrequency energy for ablation under three-dimensional electroanatomical mapping (EnSite™ NavX™) merged with three-dimensional spiral computed tomography (CT). All patients in both groups underwent a circumferential PVI. After the PVI, bidirectional block was confirmed in all patients. An extra-PV ablation was performed based on the virtual DF mapping in the V-DF group and at the operator's discretion in the E-PVI group. Examples of determining and the ablation of the CUVIA-AF 2 target in three patients in each randomized group are illustrated in Figure 3. We ablated the DF areas marked on the CT-merged 3D electroanatomical map using the focal ablation technique, and not circumferential isolation. We delivered 40–50 W of RF energy for 10–15 s, but used a reduced power and temperature on the posterior side of the LA or LA appendage. DFs located in the LA appendage were mainly at the ostium. After the protocol-based ablation, the procedure ended when no immediate recurrence of AF was observed within 10 min after cardioversion with an isoproterenol infusion (5–10 μg/min depending on ß-blocker use; target sinus heart rate of 120 bpm; AF induction by a ramp pacing cycle length of 120 ms). If further AF triggers or frequent unifocal atrial premature beats were observed under the isoproterenol effect, extra-PV foci were ablated as much as possible. If there were unmappable repetitive AF triggers at the end of the procedure, the operators targeted the potential area of the extra-PV triggers by a linear or CFAE ablation to achieve the endpoint of the protocol.

### Post-ablation Management and Follow-Up

We tried to discharge the patients without AADs except for those who had recurrent extra-PV triggers after the AFCA procedure, symptomatic frequent atrial premature beats, non-sustained atrial tachycardia (AT), or early recurrence of AF on telemetry during the admission period (AAD use 34.7% at discharge). Patients visited the outpatient clinic regularly at 1, 3, 6, and 12 months and then every 6 months thereafter or whenever symptoms occurred after AFCA. All patients underwent electrocardiography (ECG) recordings during every visit and 24-h Holter recordings at 3 and 6 months and then every 6 months thereafter, according to the protocol (25). Holter monitoring or event monitor recordings were obtained when patients reported symptoms of palpitations suggestive of an arrhythmia recurrence. A Holter analysis and adjudication were conducted by an individual blinded to the study group assignment. AF recurrence was defined as any episode of AF or AT of at least 30 s in duration. Any ECG documentation of AF recurrence within a 6-month blanking period was diagnosed as an early recurrence, and AF recurrence occurring more than 6 months after the procedure was diagnosed as a case of clinical recurrence. The primary study endpoint was the freedom from documented episodes of AF or AT lasting longer than 30 s and occurring after a 3-month blanking period after a single ablation procedure. Secondary endpoints were the periprocedural complication rate and responses to AADs or electrical cardioversion rates after postprocedural recurrence.

### Statistical Analysis

The continuous variables were compared using Student's *t*-tests, while categorical variables were compared using chi-squared or Fisher's exact tests, as appropriate. The primary endpoint was the freedom from any atrial arrhythmias during the follow-up period after a 3-month blanking period. The time to recurrence and rate of arrhythmia-free survival were assessed by a Kaplan–Meier analysis, with differences calculated using log-rank tests. To assess the factors associated with post-AFCA clinical recurrence of AF, we performed a Cox proportional-hazards model regression analysis. For all variables, *P*-values of < 0.05 were considered to be statistically significant. All statistical analyses were performed using the IBM SPSS Statistics for Windows version 26.0 software program (IBM Corp., Armonk, NY, USA).

## Results

### Patient Characteristics

Among a total of 222 patients with AAD-resistant symptomatic PeAF undergoing catheter ablation, 52 (23.4%) were excluded due to failed internal cardioversion or three episodes of recurrent AF re-initiated during paced atrial substrate mapping, which provided the mandatory electrophysiologic data for our realistic computation modeling (Figure 1). The characteristics of the study participants are summarized in Table 1. There were 87 patients included in the V-DF group and 83 included in the E-PVI group. The two ablation groups were well-balanced in terms of the baseline demographics. The mean age was 59 years, 70.6% of the study population was male, and the proportion of longstanding PeAF was 62.4%. The mean AF duration and CHA_2_DS_2_-VASc scores were 40.2 months and 2.1 ± 1.6 points, respectively. The mean LA diameter was 44.8 ± 5.5 mm. No significant difference was found regarding comorbidities between groups (*P* = NS).

**Table 1:** Baseline and procedure-related characteristics and rhythm outcomes of the study participants.

	**Overall**	**V-DF**	**E-PVI**	***P-*value**
	**(*n* = 170)**	**(*n* = 87)**	**(*n* = 83)**	
Age, years	59.2 ± 11.3	58.3 ± 11.5	60.2 ± 11.1	0.269
Male, *n* (%)	120 (70.6)	67 (77.0)	53 (63.9)	0.060
Longstanding persistent AF, *n* (%)	106 (62.4)	52 (59.8)	54 (65.1)	0.528
AF duration, (months)	40.2 ± 41.7	36.7 ± 38.8	43.9 ± 44.5	0.305
**Comorbidities**, ***n*** **(%)**			
Heart failure	40 (23.5)	22 (25.3)	18 (21.7)	0.580
Hypertension	93 (54.7)	51 (58.6)	42 (50.6)	0.294
Diabetes mellitus	39 (22.9)	19 (21.8)	20 (24.1)	0.726
Stroke	27 (15.9)	13 (14.9)	14 (16.9)	0.731
Vascular disease	13 (7.6)	9 (10.3)	4 (4.8)	0.175
CHA2DS2-VASc score	2.1 ± 1.6	2.1 ± 1.5	2.1 ± 1.6	0.719
**Echocardiographic parameters**			
LA dimension, mm	44.8 ± 5.5	44.9 ± 5.5	44.7 ± 5.5	0.777
LA volume index, ml/m2	40.6 ± 12.9	40.1 ± 13.2	41.0 ± 12.6	0.655
LV ejection fraction, %	60.1 ± 8.3	60.0 ± 7.6	60.1 ± 9.0	0.972
E/Em	10.3 ± 4.4	10.1 ± 4.6	10.4 ± 4.2	0.723
LVEDD, mm	49.7 ± 4.6	50.3 ± 4.8	49.0 ± 4.3	0.069
LVMI, g/m2	94.8 ± 21.7	95.2 ± 24.3	94.3 ± 18.7	0.777
Procedure time, min	166.8 ± 48.8	169.2 ± 43.5	164.3 ± 53.9	0.513
Ablation time, s	2989.4 ± 1067.0	3000.0 ± 958.0	2978.0 ± 1177.5	0.894
**Ablation lesions**, ***n*** **(%)**			
CPVI	170 (100)	87 (100)	83 (100)	–
Cavotricuspid isthmus line	170 (100)	87 (100)	83 (100)	–
Posterior wall isolation	8 (4.7)	3 (3.4)	5 (6.0)	0.489
Anterior line	5 (2.9)	3 (3.4)	2 (2.4)	0.689
Left lateral isthmus line	1 (0.6)	0 (0)	1 (1.2)	0.488
CFAE	2 (1.2)	1 (1.1)	1 (1.0)	0.973
Extra-PV trigger ablation, *n* (%)	4 (2.4)	1 (1.1)	3 (3.6)	0.359
Complications	8 (4.7)	3 (3.4)	5 (6.0)	0.489
Pericardial effusion	1	1	0	
PV stenosis	1	0	1	
Pericarditis	2	1	1	
Others^†^	4	1	3	
**Rhythm outcomes**			
Follow-up duration (months)	16.3 ± 5.3	16.4 ± 5.6	16.3 ± 5.3	0.903
**Post-ABL medication**			
ACEi, or ARB, *n* (%)	58 (34.1)	33 (37.9)	25 (30.1)	0.283
Beta blocker, *n* (%)	82 (48.2)	44 (50.6)	38 (45.8)	0.532
Statin, *n* (%)	63 (37.1)	35 (40.2)	28 (33.7)	0.381
**AAD use**			
At discharge, *n* (%)	59 (34.7)	29 (33.3)	30 (36.1)	0.749
After 3 months, *n* (%)	73 (42.9)	32 (36.8)	41 (49.4)	0.121
At the final follow-up, *n* (%)	53 (31.2)	19 (21.8)	34 (41.0)	0.008
Early recurrence types, *n* (%)	72 (42.4)	32 (36.8)	40 (48.2)	0.132
Clinical recurrence, *n* (%)	57 (33.5)	22 (25.3)	35 (42.2)	0.023
Recurrence type, AT, *n* (% in recur)	2 (3.5)	2 (9.1)	0	0.145
Cardioversion, *n* (% in recur/% overall)	39 (40.4/22.9)	17 (40.9/19.5)	22 (40.0/26.5)	0.362
Single-procedure success, overall, *n* (%)	113 (66.5)	65 (74.7)	48 (57.8)	0.023
Final sinus rhythm, overall, *n* (%)	142 (83.5)	81 (93.1)	61 (73.5)	0.001
Final sinus rhythm without AADs, *n* (%)	105/170 (61.8)	65/87 (74.7)	40/83 (48.2)	<0.001

*AF, atrial fibrillation; LA, left atrium; LV, left ventricle; E/Em, mitral inflow velocity/mitral annulus tissue velocity; LVEDD, LV end-diastolic diameter; LVMI, LV mass index; CFAE, complex fractionated atrial electrogram; CPVI, circumferential pulmonary vein isolation; PV, pulmonary vein; AAD, antiarrhythmic drug; ABL, ablation; ACEi, angiotensin-converting enzyme inhibitor; ARB, angiotensin receptor blocker; AT, atrial tachycardia*.

† *Other complications: sinus node dysfunction, TBS, severe hypotension, puncture site bleeding*.

### Procedural Characteristics

The procedural results and complications are summarized in Table 1. The total procedure (*P* = 0.513) and ablation (*P* = 0.894) times did not differ between the two groups. Circumferential PVI and cavotricuspid isthmus ablation were successfully conducted in all patients. We analyzed the locations of the highest 10% DF in both groups (Supplementary Table 2). The highest DF areas were commonly found in the PVs (16.5%, Figure 3A), left atrial appendage (11.2%), or roof (10.0%). Multiple DF areas were found in 17.6% of patients and we ablated any extra-PV DF areas in the V-DF ablation group (Figure 3B) but not in the E-PVI group (Figure 3C). In the V-DF group, 37.6% of the patients (25/87) did not undergo a V-DF ablation because the V-DF was located inside the PVs (20.7%) or there was the absence of any presentation of DFs in the induced virtual ATs (16.9%). In the E-PVI group, an additional posterior box isolation and anterior linear ablation were conducted in 6.0 and 2.4% of the patients, respectively, at the operators' discretion, mostly due to repetitive immediate triggering of AF under an isoproterenol provocation. We conducted linear ablation only in the minority of the patients (4.6% for V-DF group vs. 6.0% for E-PVI group, *p* = 0.742), and the bidirectional block rates did not differ between the two groups (55.6% for V-DF group vs. 44.4% for E-PVI group, *p* = 0.099). The complication rates also did not significantly differ between the groups (3.4 vs. 6.0%; *P* = 0.489). No major thromboembolic complications, including strokes, occurred in either group (Table 1).

### Primary Outcomes

**Figure 4:**
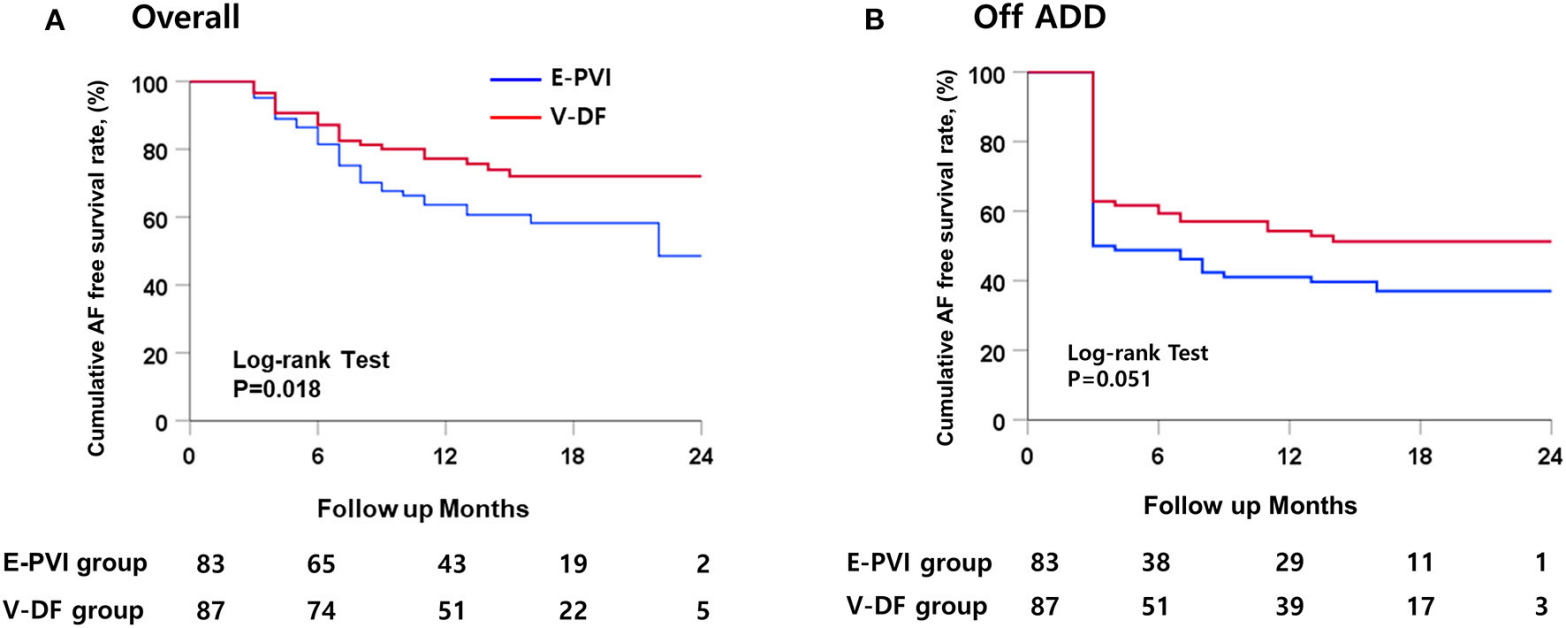
Kaplan–Meier curves for the cumulative AF-free survival. **(A)** Overall patients. **(B)** Patients maintaining AAD use after catheter ablation.

During the 16.3 ± 5.3 months of follow-up, the early recurrence rates within 3 months of the AFCA did not differ (36.8 vs. 48.2%; *P* = 0.132), but the clinical recurrence rate was significantly lower in the V-DF group than E-PVI group (25.3 vs. 42.2%; *P* = 0.023, Table 1). A Kaplan–Meier analysis revealed a significantly lower clinical recurrence rate in the V-DF group than E-PVI group overall (*P* = 0.018, log-rank, Figure 4A). The rate of freedom from AF with off-AAD tended to be higher in the V-DF group (*P* = 0.051, log-rank, Figure 4B).

### Secondary Outcomes and Subgroup Analysis

The AAD prescription rates did not significantly differ between the two groups at discharge (33.3 vs. 36.1%; *P* = 0.749) or 3 months after the procedure (36.8 vs. 49.4%; *P* = 0.121). However, the V-DF group demonstrated a significantly lower rate of AAD prescriptions than that of the E-PVI group at the final follow-up (21.8 vs. 41.0%; *P* = 0.008, Table 1). The overall single-procedure success rate was significantly higher in the V-DF group (74.7%) than the E-PVI group (57.8%; *P* = 0.023, Table 1). A multivariate Cox regression analysis showed that the V-DF ablation was independently associated with a low clinical recurrence after AFCA (hazard ratio 0.51, 95% confidence interval 0.30–0.88, *P* = 0.016, Table 2).

**Table 2:** Cox regression analysis for clinical recurrence after catheter ablation.

	**Univariate**		**Multivariate**	
	**HR (95% CI)**	***P*-value**	**HR (95% CI)**	***P*-value**
Age, years	1.01 (0.97–1.04)	0.578	1.00 (0.98–1.03)	0.740
Male, *n* (%)	1.09 (0.63–1.90)	0.756	1.35 (0.76–2.40)	0.305
AF duration	1.00 (0.99–1.01)	0.595		
Heart failure	1.01 (0.45–2.27)	0.974		
Hypertension	1.16 (0.57–2.35)	0.681		
Diabetes mellitus	1.10 (0.49–2.50)	0.815		
Stroke	1.26 (0.55–2.88)	0.581		
CHA_2_DS_2_-VASc score	1.05 (0.89–1.23)	0.601	1.09 (0.88–1.36)	0.423
LA dimension, mm	1.01 (0.96–1.11)	0.666	1.01 (0.96–1.07)	0.654
LVEF, %	0.99 (0.95–1.03)	0.523		
E/Em	0.96 (0.86–1.07)	0.474		
Computational virtual-guided	0.54 (0.31–0.92)	0.022	0.51 (0.30–0.88)	0.016

*AF, atrial fibrillation; CHA2DS2-VASc, Congestive heart failure, Hypertension, Age (>65, 1 point; >75, 2 points), Diabetes, previous Stroke/transient ischemic attack (2 points); LA, left atrium; LV, left ventricular ejection fraction; E/Em, mitral inflow velocity/mitral annulus tissue velocity*.

**Figure 5:**
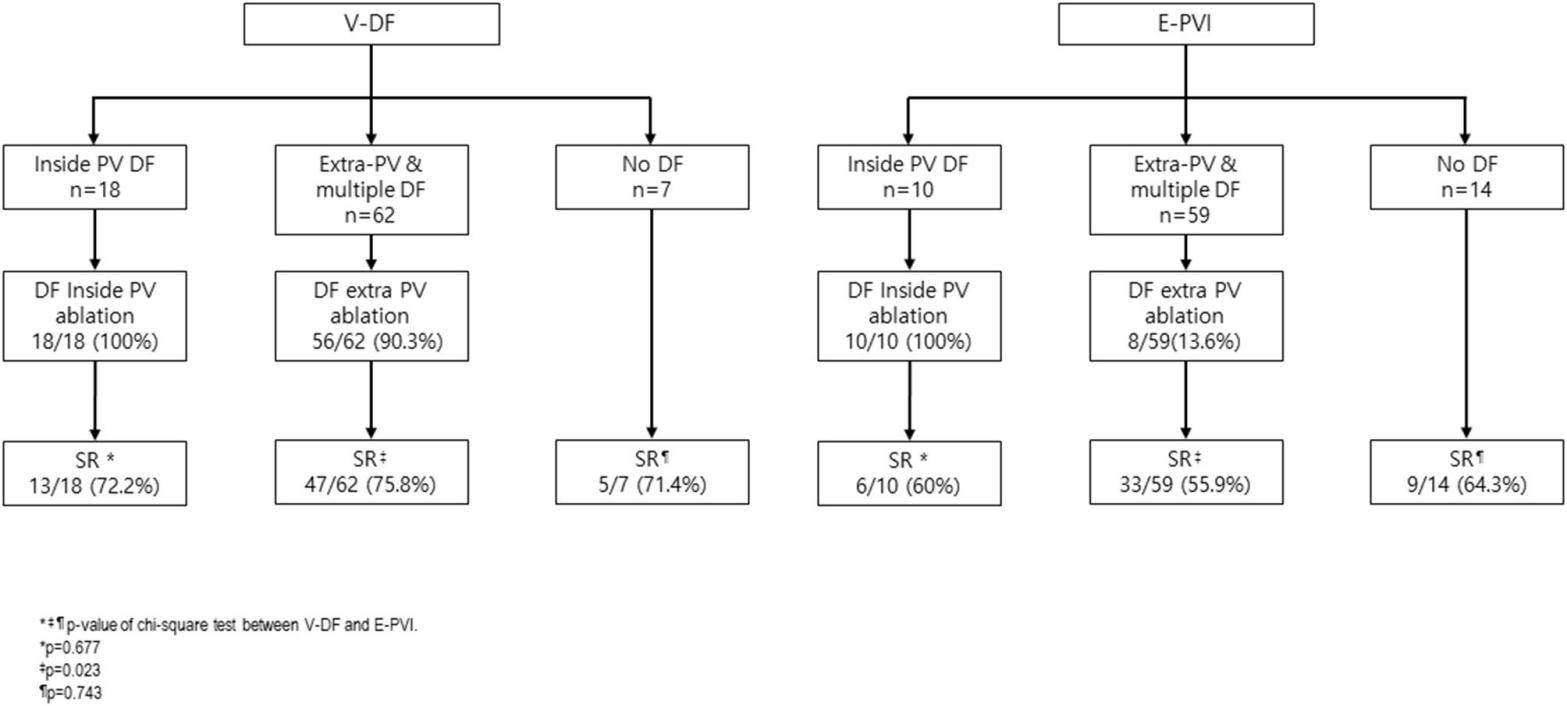
Procedure outcome according to DF location and ablation.

In sub-analysis of DF extra PV ablation, V-DF group showed better rhythm outcome compared to E-PVI group (24.2% recurrence for V-DF vs. 44.1% recurrence for the E-PVI; *p* = 0.023, Figure 5) Totally, we conducted the empirical extra-PV ablation in 11 out of 83 patients in the E-PVI group. Although the result of the DF map was not noticed to the operators in the E-PVI group, the DF site and empirical extra-PV ablation site matched in 2 out of 11 patients in the retrospective analysis (Supplementary Table 3).

Out of 57 patients with clinical recurrence, 55 experienced AF recurrence and two experienced AT recurrence (Table 1). Among the patients with clinical recurrence, the proportion of AT [9.1% (2/22) vs. 0% (0/35); *P* = 0.145] and the cardioversion rates [40.9% (9/22) vs. 40.0% (14/35), *P* = 0.590] did not differ between the V-DF and E-PVI groups. Overall, 19.5% (17/87) of the V-DF group and 26.5% (22/83) of the E-PVI group underwent cardioversion to control AAD-resistant recurring atrial arrhythmias (*P* = 0.362). Finally, the proportions of patients who remained in sinus rhythm were 93.1% in the V-DF group and 73.5% in the E-PVI group (*P* = 0.001), respectively, while those who remained in sinus rhythm without AADs totaled 74.7% in the V-DF group and 48.2% in the E-PVI group (*P* < 0.001, Table 1).

## Discussion

In this prospective RCT comparing real-time computational modeling–guided AFCA and empirical AFCA in patients with PeAF, the intraprocedural virtual DF mapping and ablation targeting DF areas improved the rhythm outcomes without increasing the procedure time or risk of complications. We demonstrated that realistic computational modeling of AF, which reflects a personalized atrial anatomy, electrophysiology, fibrosis, and fiber orientation, is feasible and effective in non-paroxysmal AF ablation.

### Heterogeneity and Unmet Needs of AF Ablation in Patients With PeAF

Since AF is a progressive disease, there are various stages of atrial remodeling in the category of PeAF. The current classification of AF is mainly determined by the duration of sustained AF, but the AF duration is often not clear in minimally symptomatic patients. Moreover, depending on the treating institution, some patients with longstanding PeAF undergo AFCA directly, while others undergo AFCA only after changing the AF to the paroxysmal type with AADs and cardioversion (6). Therefore, the current classification does not accurately reflect the pathophysiology of AF, and it is rather natural that prior attempts to conduct a uniform empirical extra-PV ablation for PeAF have failed. All the RCTs and meta-analyses that compared the PVI and other empirical extra-PV ablation procedures, such as the STAR-AF2 (comparing linear ablation and electrogram-guided ablation) and rotor ablation trials, failed to demonstrate the clinical usefulness of additional empirical extra-PV ablation lesions in patients with PeAF (7–9). As for the extra-PV ablation protocol that has been proven effective so far, mapping and ablation of extra-PV triggers after an isoproterenol provocation was the only approach that worked. However, mapping techniques available for provoked extra-PV triggers are still limited (26). In this study, we conducted an isoproterenol provocation and extra-PV trigger mapping and ablation in all patients included.

### Computational Modeling-Guided AF Ablation

Since Moe et al. (27) published on the subject of human atrial cell modeling in 1964, computational modeling has continued to evolve and innovate, playing an important part in basic electrophysiology research (14, 28). In recent years, sophisticated modeling is possible by reflecting the cardiac magnetic resonance imaging (MRI) late-gadolinium enhancement (29). Nevertheless, the biggest obstacle that exists in applying AF computational modeling to clinical medicine has been the high computational burden. Recently, however, this limitation has been overcome by a parallel computing technology (30), and MRI-based computational modeling has become technically feasible for arrhythmia interventions (29). Simulation-guided AFCA provides precision medical care that reflects the patient's personalized anatomy, histology, and electrophysiological characteristics (31).

The CUVIA-AF 1 trial was the only prospective RCT of computational modeling-guided AFCA published to date (10, 11). Kim et al. (10) conducted a preprocedural virtual ablation to test the optimal linear lesion set using AF modeling (CUVIA version 1.0) (32), which reflected the patient-specific atrial anatomy, and adopted the best lesion set during the clinical AFCA procedure, which was superior to the empirical ablation in patients with PeAF. In the current study, CUVIA version 2.5, which further improved the computational power and calculation efficiency (12), was used to test the effects of the intraprocedural AF driver mapping and ablation in a prospective RCT. After applying the CT-based anatomy, personalized electrophysiology, fibrosis, and fiber orientation inferred from a clinical electroanatomical map, the DF target information was successfully calculated and provided to the operator within 30–40 min while performing the PVI. DF ablation showed the better outcome for AF termination or defragmentation rates as compared to the CFAEs, phase singularities, or Shannon entropy in the previous virtual ablation study (13). It has been reported that a spectral analysis and frequency mapping identify localized sites of high-frequency activity during AF in humans with different distributions in non-paroxysmal AF (8, 11, 33). We targeted the DF sites as extra-PV AF drivers because the AF termination rate was higher after a DF ablation than other parameters representing rotational reentries in the previous simulation study (13). Unlike as seen in this CUVIA-AF2 trial, the RADAR-AF trial (34), which involved an extra-PV DF target ablation, reported negative outcomes as compared to a PVI alone. However, the mapping method in the RADAR-AF trial apparently differs from the entire-chamber DF mapping protocol used in this study. We acquired electrical data by a point-by-point map using a multi-electrode catheter during high right atrial pacing. We did not generate voltage maps in the AF state because AF drivers or rotational reentries meander during AF maintenance. Therefore, we localized virtual DF sites during the virtual AF state after integrating the clinical voltage and activation map to the computational model. The virtual AF analyzed in this study was an entire chamber map, which differs from the point-by-point sequential AF map in the RADAR trial.

### Hurdles to Overcome and the Future Direction

Although AF modeling has advanced to the point of intraprocedural simulation and mapping, there are still challenges ahead. First, invasive mapping data are required as an important reference for fibrosis, conduction velocity, and the fiber orientation (12). Second, the dropout rate was 23.4% during the acquisition of clinical paced atrial substrate maps because of cardioversion failure or recurrent re-initiation of AF. Third, atrial epi- and endocardial dissociation of the activation have to be reflected (35). We improved CUVIA version 3.0 (36) to a stage at which the atrial wall thickness can be applied, but the program still needs to overcome the increased computational burden. Fourth, most of the current modeling is chamber-centric mapping as it is hard to clearly determine the interatrial conduction pattern using medical images or clinical electroanatomical maps. Fifth, AF is a multifactorial disease and future AF modeling requires cardiac autonomic nerve activity, epicardial fat, or metabolic factors in order to apply various pathophysiologies (37). Sixth, it needs considering the possibility in misinterpretation of the results about secondary outcomes and subgroup analysis in this study, because we did not conduct control and adjustment for multiple hypothesis test in the secondary outcomes and subgroup analysis. Seventh, we conducted extra-PV ablation in 11 out of 83 patients in the E-PVI group to achieve the ablation end-point. Finally, it is necessary to expand the clinical application fields of AF modeling—for example, by considering virtual AADs—as well as arrhythmia intervention. With the application of artificial intelligence and the innovation of the hardware, we will overcome such hurdles one by one, however, we will be challenged each time by the computational burden and time.

## Conclusion

In this prospective RCT comparing the real-time computational modeling–guided AFCA and empirical AFCA, intraprocedural virtual DF mapping and ablation targeting DF areas improved the rhythm outcomes without increasing the procedure time or risk of complications among the patients with PeAF. We proved that realistic computational modeling of AF, which reflects a personalized atrial anatomy, electrophysiology, fibrosis, and fiber orientation, is feasible and effective in non-paroxysmal AF ablation.

## Data Availability Statement

The raw data supporting the conclusions of this article will be made available by the authors, without undue reservation.

## Ethics Statement

The studies involving human participants were reviewed and approved by the Institutional Review Board of Yonsei University Health System, Inha University College of Medicine and Inha University Hospital, and Ewha Womans University. The patients/participants provided their written informed consent to participate in this study.

## Author Contributions

Y-SB and O-SK contributed to writing the manuscript and data analysis. O-SK, BL, and S-YY developed the computational modeling and participate in simulation procedures. J-WP contributed to the data analysis and the manuscript revision. HY, T-HK, J-SU, BJ, D-HK, M-HL, JP, and H-NP conducted a multicenter clinical trial. H-NP and JP controlled all the *in silico* and clinical studies, manuscript preparation, and funding. All authors contributed to the article and approved the submitted version.

## Funding

This work was supported by grants (HI19C0114 and HI21C0011) from the Ministry of Health and Welfare and grants (NRF-2019R1C1C1009075 and NRF-2020R1A2B01001695) from the Basic Science Research Program run by the National Research Foundation of Korea (NRF), which is funded by the Ministry of Science, ICT & Future Planning (MSIP).

## Conflict of Interest

The authors declare that the research was conducted in the absence of any commercial or financial relationships that could be construed as a potential conflict of interest.

## Publisher's Note

All claims expressed in this article are solely those of the authors and do not necessarily represent those of their affiliated organizations, or those of the publisher, the editors and the reviewers. Any product that may be evaluated in this article, or claim that may be made by its manufacturer, is not guaranteed or endorsed by the publisher.
