# Supplementary material for: Clinical Outcomes of Computational Virtual Mapping-Guided Catheter Ablation in Patients With Persistent Atrial Fibrillation: A Multicenter Prospective Randomized Clinical Trial
Source: Front Cardiovasc Med. 2021 Dec 8;8:772665. doi: 10.3389/fcvm.2021.772665 (PMC8692944; doi:10.3389/fcvm.2021.772665)
Supplement: Supplementary file 1 [file Data_Sheet_1.docx]

**Supplementary Table 1.** **Ion current changes in CUVIA-AF2**

|  | **Sinus rhythm* (%)** | **AF (%)** | **Percent change (%)** |  |
| --- | --- | --- | --- | --- |
| **I_Na_** | 111 | 100 | -10 |  |
| **I_K1_** | 95 | 200 | +111 |  |
| **I_to_** | 117 | 35 | -70 |  |
| **I_Kr_** | 120 | 100 | -17 |  |
| **I_CaL_** | 150 | 45 | -70 |  |
| **I_Kur_** | 100 | 50 | -50 |  |
| **I_Ks_** | 160 | 150 | -6 |  |
| **INaCa (Max)** | 155 | 155 | 0 |  |
| **INaK (Max)** | 100 | 100 | 0 |  |
| **Iup (Max)** | 100 | 100 | 0 |  |
| **Krel** | 100 | 100 | 0 |  |
| **Caup (Max)** | 125 | 100 | -20 |  |
| **ACh** | 100 | 100 | 0 |  |
| * refers to the Courtemanche Ramirez Nattel atrial model. | | | | |

**Supplementary Table 2. Anatomical distribution of the highest 10% DF areas**

|  | **Overall** | **V-DF** | **E-PVI** | **P-value** |
| --- | --- | --- | --- | --- |
|  | **(n=170)** | **(n=87)** | **(n=83)** |  |
| PV, n (%) | 28 (16.5) | 18 (20.7) | 10 (12.0) | 0.151 |
| LSPV, n (%) | 6 (3.5) | 5 (5.7) | 1 (1.2) |  |
| LIPV, n (%) | 7 (4.1) | 4 (4.6) | 3 (3.6) |  |
| RSPV, n (%) | 10 (5.9) | 7 (8.0) | 3 (3.6) |  |
| RIPV, n (%) | 5 (2.9) | 2 (2.3) | 3 (3.6) |  |
| Extra-PV, n (%) | 91 (53.5) | 48 (55.2) | 43 (51.8) | 0.759 |
| Septum, n (%) | 15 (8.8) | 5 (5.7) | 10 (12.0) |  |
| Anterior wall, n (%) | 15 (8.8) | 7 (8.0) | 8 (9.6) |  |
| LAA, n (%) | 19 (11.2) | 9(10.3) | 10 (12.0) |  |
| Peri-mitral area, n (%) | 12 (7.1) | 5 (5.7) | 7 (8.4) |  |
| Posterior wall, n (%) | 13 (7.6) | 9 (10.3) | 4 (4.8) |  |
| Roof, n (%) | 17 (10.0) | 13 (14.9) | 4 (4.8) |  |
| Multiple DF, n (%) | 30 (17.6) | 14 (16.1) | 16 (16.3) | 0.688 |
| No DF, n (%) | 21 (12.4) | 7 (8.0) | 14 (16.9) | 0.103 |
| DF, dominant frequency; LAA, left atrial appendage; PV, pulmonary vein. | | | | |

**Supplementary Table 3. Comparison between empirical extra-PVI ablation sites and virtual DF sites in the E-PVI**

| **Cases No.** | **Empirical extra-PV ablation site** | **Virtual DF site** | **Extra-PV ablation site overlapping with DF sites** |
| --- | --- | --- | --- |
| 1 | posterior wall isolation | roof | Yes |
| 2 | posterior wall isolation | anterior wall | No |
| 3 | posterior wall isolation | anterior wall | No |
| 4 | posterior wall isolation | peri-mitral area | No |
| 5 | posterior wall isolation | left atrial appendage | No |
| 6 | anterior line | roof | No |
| 7 | anterior line, left anterior wall* | no DF | No |
| 8 | left lateral isthmus line | anterior wall | No |
| 9 | CFAE | septum | Yes |
| 10 | low septal trigger* | multiple | No |
| 11 | high septal trigger* | low septum | No |

*extra-foci triggered by isoproterenol test after protocol ablation

CFAE, complex fractionated atrial electrogram; DF, dominant frequency
